# A Fast, Efficient Domain Adaptation Technique for Cross-Domain Electroencephalography(EEG)-Based Emotion Recognition

**DOI:** 10.3390/s17051014

**Published:** 2017-05-03

**Authors:** Xin Chai, Qisong Wang, Yongping Zhao, Yongqiang Li, Dan Liu, Xin Liu, Ou Bai

**Affiliations:** 1 School of Electrical Engineering and Automation, Harbin Institute of Technology, Harbin 150001, China; 11b901011@hit.edu.cn (X.C.); zhaoyp2590@hit.edu.cn (Y.Z.); liyongqiang@hit.edu.cn (Y.L.); liudan@hit.edu.cn (D.L.); 2 Department of Traffic Information and Control Engineering, School of Transportation Science and Engineering, Harbin Institute of Technology, Harbin 150001, China; xinliu@hit.edu.cn; 3 Department of Electrical and Computer Engineering, Florida International University, Miami, FL 33199, USA; obai@fiu.edu

**Keywords:** Electroencephalography (EEG), emotion recognition, domain adaptation

## Abstract

Electroencephalography (EEG)-based emotion recognition is an important element in psychiatric health diagnosis for patients. However, the underlying EEG sensor signals are always non-stationary if they are sampled from different experimental sessions or subjects. This results in the deterioration of the classification performance. Domain adaptation methods offer an effective way to reduce the discrepancy of marginal distribution. However, for EEG sensor signals, both marginal and conditional distributions may be mismatched. In addition, the existing domain adaptation strategies always require a high level of additional computation. To address this problem, a novel strategy named adaptive subspace feature matching (ASFM) is proposed in this paper in order to integrate both the marginal and conditional distributions within a unified framework (without any labeled samples from target subjects). Specifically, we develop a linear transformation function which matches the marginal distributions of the source and target subspaces without a regularization term. This significantly decreases the time complexity of our domain adaptation procedure. As a result, both marginal and conditional distribution discrepancies between the source domain and unlabeled target domain can be reduced, and logistic regression (LR) can be applied to the new source domain in order to train a classifier for use in the target domain, since the aligned source domain follows a distribution which is similar to that of the target domain. We compare our ASFM method with six typical approaches using a public EEG dataset with three affective states: positive, neutral, and negative. Both offline and online evaluations were performed. The subject-to-subject offline experimental results demonstrate that our component achieves a mean accuracy and standard deviation of 80.46% and 6.84%, respectively, as compared with a state-of-the-art method, the subspace alignment auto-encoder (SAAE), which achieves values of 77.88% and 7.33% on average, respectively. For the online analysis, the average classification accuracy and standard deviation of ASFM in the subject-to-subject evaluation for all the 15 subjects in a dataset was 75.11% and 7.65%, respectively, gaining a significant performance improvement compared to the best baseline LR which achieves 56.38% and 7.48%, respectively. The experimental results confirm the effectiveness of the proposed method relative to state-of-the-art methods. Moreover, computational efficiency of the proposed ASFM method is much better than standard domain adaptation; if the numbers of training samples and test samples are controlled within certain range, it is suitable for real-time classification. It can be concluded that ASFM is a useful and effective tool for decreasing domain discrepancy and reducing performance degradation across subjects and sessions in the field of EEG-based emotion recognition.

## 1. Introduction

Affective computing, which integrates a user’s emotions into human–computer interaction, has attracted much research attention in recent years. Emotion recognition, one of the most important elements in affective computing, aims to create affective user interfaces between humans and computers and to help in detecting psychiatric health in patients. A variety of measures such as facial expression, speech, electrocardiograph (ECG), electromyogram (EMG) and Electroencephalography (EEG) [1,2,3,4,5,6] have been used for emotion recognition; of these, methods based on EEG signals are attracting increasing levels of attention due to their objective evaluation and immediate responses to emotional stimuli [7,8,9]. In general, to improve the spatial resolution of EEG, 64 or more sensor electrodes are needed in order to record EEG signals [10].

The classification of EEG signals requires the extraction of useful features using an adequate transformation of the raw EEG signal [11,12,13]; following this, various types of classifiers such as a support vector machine (SVM) and linear regression (LR) can be implemented with multiclass EEG signal classification. However, it is worth mentioning that the above classification strategies involve the training and testing of the classifier for each subject. Thus far, it has remained difficult to classify EEG patterns across subjects with a conventional classifier because of the variability between subjects and the high levels of non-stationarity of EEG sensor signals [14]. Studies in the literature [15,16] have proven factors causing non-stationarity. In EEG-based emotion recognition systems, the distribution between the training and testing samples may be mismatched if they are sampled from different experimental sessions or subjects. For a single subject on a given day, well-trained classifiers can perform accurately; however, the performance is usually degraded on subsequent days when using the same experimental setup. In across-subject classification problems, the training and test data are usually sampled from different probability distributions. Thus far, most studies have focused on selecting features or channels [17,18,19,20] and have neglected to consider the distribution discrepancy between subjects.

Recently, domain adaptation learning techniques, which have been proven to be effective in reducing the difference in distributions between domains, have been vigorously investigated for cross-domain knowledge adaptation problems. Domain adaptation is typically aimed at identifying common representations across training and test domains and building classifiers which are robust to mismatched distributions, assuming that the input distributions of training and testing sessions are different, while the conditional distribution of output given input remains unchanged.

To overcome the performance decrease caused by the non-stationarity of EEG sensor signals, several domain adaptation methods have been proposed [21,22,23]. In [15], Sugiyama et al. applied a covariate shift adaptation method to a brain-computer interface (BCI) competition dataset to analyze non-stationary EEG signals in different sessions. They assumed that although the marginal distribution between the subjects or sessions was mismatched, the decision rule remained constant. To correct the covariate shifts, they utilized the Kullback–Leibler importance estimation procedure to re-weight the training data and correct the difference between different subjects or sessions. More widely used methods attempt to employ transformation to project the source and target domain onto a subspace. In previous work [24], a new domain adaptation method known as transfer component analysis (TCA) was proposed to learn some transfer components across domains in a reproducing kernel Hilbert space (RKHS) using the maximum mean discrepancy (MMD) constraint [25]. In this subspace, the marginal distributions of data in different domains are similar to each other. As a result, any supervised method can be applied to the new source domain to train a classifier for use in the target domain. On this basis [26], a novel transfer joint matching (TJM) approach has been put forward in the literature for model feature matching and instance reweighting in a unified framework. Specifically, TJM aims to construct a new feature representation that is invariant to both the distribution difference and the irrelevant instances. In [27], a reconstruction strategy based on sparse coding was used in an attempt to construct robust sparse representations of both source and target data to obtain a well-aligned feature space, in order to minimize the distribution divergence between the training and test data. The authors incorporate this criterion into the objective function of sparse coding to make the new representations robust to the distribution discrepancy. In addition, auto-encoder networks, which can learn a common representation across training and test domains, have been successfully applied for reducing the cross-domain discrepancy by utilizing the deep structures in the field, for example face recognition and speech recognition [28,29,30].

In literature [31] a new EEG feature selection approach was developed: transfer recursive feature elimination (T-RFE), for cross-subject emotion classification. The effectiveness of the T-RFE algorithm for such cross-subject emotion classification paradigm is validated by a database for emotion analysis using physiological signals (DEAP) [32], and outperforms several recent reported works on the same database. The authors of [31] proposed a semi-supervised strategy by utilizing a validating set with the emotion class labels rom the target domain. This is a successful attempt to handle the cross-subject emotion classification in the DEAP database. The authors of [33] proposed an adaptive stacked denoising auto-encoder (SDAE) to tackle cross-session mental workload levels classification tasks using EEG. The algorithm of the adaptive SDAE consists of two steps: (1) initialization of the deep model; and (2) the adaptive classification by iteratively tuning the weights between the adaptive layer and the previous layer of the network. The results indicate a higher performance of the adaptive SDAE in dealing with the cross-session EEG features. In [22], Zheng et al. mainly explored two types of subject-to-subject transfer approaches without labeled target data. One is to exploit the shared structure between training and test data. The other is to train multiple individual classifiers on source subjects and transfer knowledge about classifier parameters to target subjects. The experimental results show a mean accuracy of 76.31% in comparison with a conventional generic classifier, with 56.73% on average. However, this approach requires that there are enough subjects in the database, and it cannot handle the session-to-session experiment. In [34], a novel strategy called the subspace alignment auto-encoder (SAAE) is proposed to combine the auto-encoder network and the subspace alignment solution in a unified framework to handle the unsupervised domain adaptation problem for EEG-based emotion recognition by transforming features into the domain-invariant subspace. SAAE exhibits better performance when compared to the state-of-the-art methods. However, for any new samples from the testing sessions, the domain-invariant feature must be relearned. This involves significant additional computation, and renders SAAE unsuitable for real-time classification.

In addition, the domain adaptation strategies mentioned above are actually a trade-off between learning domain-invariant feature and retaining information in both source and target domains. Transforming the data to an intermediate common shared subspace may lead to information loss in both source and target domains. Moreover, these domain adaptation methods learn common representations by assuming that the marginal distribution of training and testing sessions are different, while the conditional distribution remains unchanged. However, for the EEG sensor signals, both marginal and conditional distributions may be mismatched due to fluctuations in user attention level and user fatigue.

**Figure 1:**
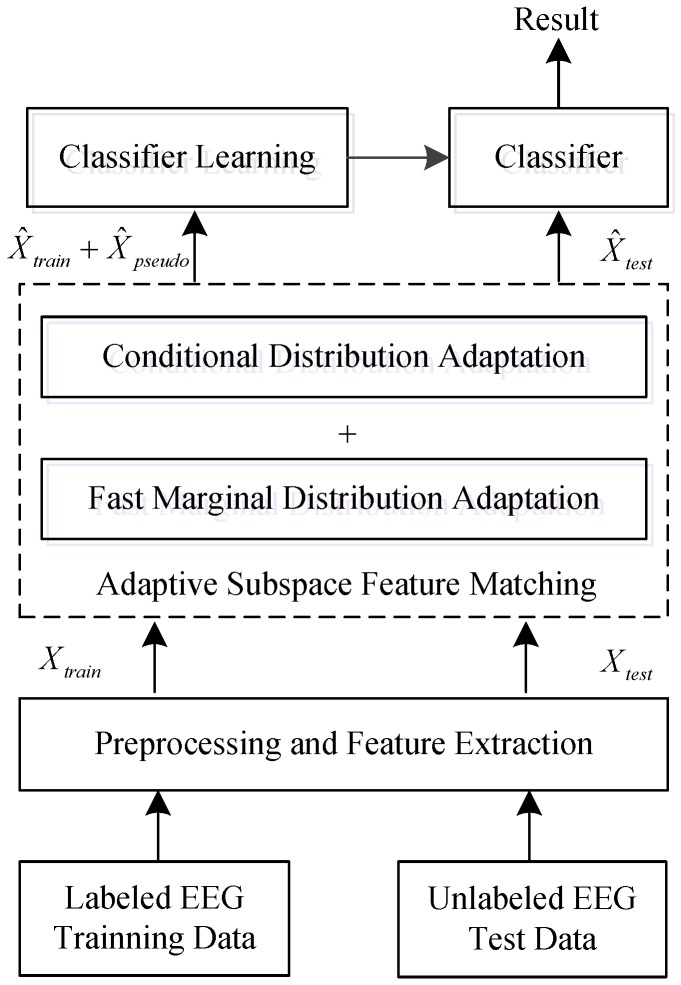
The flowchart of the Electroencephalography (EEG) emotion recognition system integrating the proposed domain adaptation method.

To solve these problems, a fast domain adaptation strategy known as adaptive subspace feature matching (ASFM) is proposed in this paper. In this method, we jointly adapt both the marginal and conditional distributions within a unified framework, as shown in Figure 1. Specifically, instead of tuning regularization parameters in the objective function like most domain adaptation methods, this approach involves learning a linear transformation function which matches the marginal distribution of the source and target subspaces without a regularization term. This means that the time complexity of our domain adaptation procedure is significantly reduced.

In this study, by modeling each channel band power as a separate feature, an efficient feature known as differential entropy (DE) [11,12] was used to measure the complexity of the EEG signals forming the input of the ASFM model. In ASFM, the *l* largest eigenvalues induced by principal component analysis (PCA) are first selected for each domain as the bases of the source and target subspaces. Then, the linear transformation matrix S is learned by minimizing the Bregman matrix divergence. This step allows us to directly move the source and target subspaces closer without unnecessary data projections. Additionally, any supervised method can be applied in the new source domain to train a classifier for classification in the target domain, as the aligned source domain follows a distribution similar to that of the target domain. In order to match the conditional distributions, we try to estimate the prediction probability for an input target sample in the aligned subspace, and move the confident predictions into the training set to adjust the conditional distributions. Note that our ASFM is an unsupervised strategy without any labeled test data.

The major contributions of this research can be summarized as follows:

1. In contrast to the conventional domain adaptation method, we combine the marginal and conditional distribution adaptation strategies in the subspace, meaning that the new feature is effective and robust for substantial distribution discrepancies;
2. Although the classifier in our proposed method needs to be updated along with the newly obtained EEG data, the computational efficiency is much better than standard domain adaptation, and it indicates the capability for the online implementation of the proposed algorithm when the number of training samples and test samples is controlled within certain range;
3. Our work is intended to provide evaluation and analysis of several state-of-the-art domain adaptation techniques in the field of pattern recognition for researchers aiming to apply these to non-stationary EEG signal classification.

The remainder of this paper is organized as follows. In Section 2, the proposed experimental design is introduced. Section 3 gives the details of the proposed ASFM method. Section 4 presents the experimental results and a comparison with state-of-the-art methods. In Section 5, a discussion and tools for analysis are given. The paper concludes in Section 6 with a summary of our study and possible future extensions.

## 2. Experiment Design

This study was performed on the publicly available emotion dataset – Shanghai Jiao Tong University (SJTU) emotion EEG dataset (SEED) (http://bcmi.sjtu.edu.cn/~seed/) [13] in order to investigate the effectiveness of the proposed domain adaptation algorithms. The SEED dataset is the best choice for evaluating the influence of distribution discrepancy both across subjects and sessions for non-stationary EEG signal classification, since each subject participating in SEED underwent the experiments for three sessions with an interval of one week or longer. In SEED, EEG signals from a total of 15 participants (eight females and seven males with an average age of 23.27) were recorded using an ESI NeuroScan system with 62-AgCl-electrode cap (channels) electrodes at a sampling rate of 1000 Hz. Each subject was exposed to stimulation from Chinese film clips (since factors related to native culture may affect elicitation in emotional experiments). For the feedback, participants were asked to assess their emotions as positive, neutral or negative immediately after watching each film clip. Participants were told to report their emotional reactions to each film clip by completing the questionnaire immediately after watching each clip. The questions are as follows: (1) what they had actually felt in response to viewing the film clip; (2) how they felt at the specific time they were watching the film clips; (3) had they watched this movie before; (4) had they understood the film clips. They also rate the intensity of subjective emotional arousal using a 5-point scale. If the target emotions were elicited, the corresponding experiment epochs were added into the SEED dataset.

**Figure 2:**
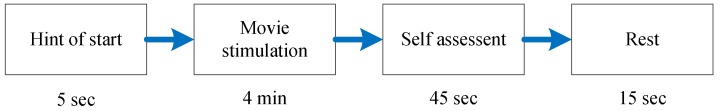
Protocol for the electroencephalography-based emotion recognition experiment (adapted from [13]).

For each participant in SEED, each positive, neutral and negative emotion has five corresponding emotional clips, and each trial lasts about four minutes. The order of film clips is arranged in advance so that two film clips targeting the same emotion are not shown consecutively. As shown in Figure 2, there is a 5-s hint before each clip and 45 s of feedback after each clip. The SEED dataset provides EEG signals which are down-sampled to 200 Hz; all of the EEG data underwent manual inspection to remove the samples which had been seriously contaminated by electromyogram (EMG) and electrooculogram (EOG). In addition, EOG signals were also recorded to mark blank artifacts. A bandpass filter was applied between 0.3 Hz to 50 Hz to preprocess the EEG data, in order to filter the noise and remove these artifacts. For each experiment, there are about 3300 clean epochs in total in each channel; each epoch lasts for 1 s without overlapping.

## 3. Proposed Method

### 3.1. Feature Extraction and Normalization

Various types of features, such as power spectral density (PSD), differential entropy (DE), differential asymmetry (DASM), rational asymmetry (RASM), and asymmetry (ASM) have been used in EEG-based emotion recognition. According to the analysis results presented in [11,12,13], a simple and efficient feature known as differential entropy (DE) shows a relatively balanced performance compared with other features. DE is defined as:

(1) $$h_{i}\left(X\right)=-\int_{-\infty }^{\infty }\frac{1}{\sqrt{2\pi \sigma^{2}}}exp\frac{{\left(x-\mu \right)}^{2}}{2\sigma^{2}}\log\frac{1}{\sqrt{2\pi \sigma^{2}}}exp\frac{{\left(x-\mu \right)}^{2}}{2\sigma^{2}}dx=\frac{1}{2}\log2\pi e\sigma^{2}$$

where the time series *X* obeys the Gauss distribution $N(\mu ,\sigma^{2})$. Obviously, the original EEG signals do not follow a certain fixed distribution. However, authors of [11,12] have proven that: after band-pass filtering in a certain band, the probability of sub-band signals meeting Gaussian distribution hypothesis is greater than 90 percent. Therefore, in this paper, the time series *X* refers to EEG signals after band-pass filtering in the five frequency bands: delta (1–3 Hz); theta (4–7 Hz); alpha (8–13 Hz); beta (14–30 Hz); and gamma (31–50 Hz). For each frequency band, we can get the corresponding time series X, then calculate corresponding DE feature following Formula Equation (1). Although DE can achieve significantly better performance, there are two difficulties with this approach. Due to the non-stationarity in the EEG sensors, the same signal may indicate different concepts under different conditions. In other words, the conditional probability may change for samples collected from different participants. Conventional DE features give equal treatment to all of the training samples; hence, samples with the same emotion but collected from different participants cannot be distinguished. In this work, a general coding scheme which takes into account the variation in participants is used to enrich the DE feature. Suppose there are *N* participants in the training set. The *N* dimensions extra feature vector of the *q*th sample $H_{q}\in R^{N}$ is defined as: $H_{q}=\left[h_{1},h_{2},...,h_{k},...h_{N}\right]$. If this sample in the training set is from the *p*th subject (*N* subjects in total), $h_{p}=1$ and is 0 otherwise. Therefore, the *k*th element $h_{k}$ in the *N* dimensions vector $H_{q}$ can be defined as:

(2) $$h_{k}=\left\{\begin{array}{cc} 1 & if\;k=p\\ 0 & otherwise \end{array}\right.$$

In this work, if there are *M* samples from the source domain and there are *N* participants in the training set, then we can augment the DE feature of the *q*th sample in the training set by concatenating the original features $x\in R^{M}$ and the extra feature vector $H_{q}$ as:

(3) $$\hat{x_{q}}=\left[\begin{array}{c} x_{q}\\ H_{q} \end{array}\right]\in R^{M+N}$$

In short, the features of conventional DE have been extended to carry more information about the background. In this study, the augmented DE was calculated using the standard five frequency bands (delta: 1–3 Hz, theta: 4–7 Hz, alpha: 8–13 Hz, beta: 14–30 Hz, gamma: 31–50 Hz). Thus, there are 325 dimensions of features in total, since each frequency-band signal has 62 channels and there are 15 participants in the SEED dataset.

### 3.2. Adaptive Subspace Feature Matching

In this study, the augmented DE feature vectors extracted from samples in both the training and test sets were used as the input to our ASFM model. In the source domain, there are ns labeled samples in *d* dimensions (d=325), denoted as $X_{S}=\left[x_{s1},x_{s2},...,x_{sns}\right]$ with their class labels $Y_{s}=\left[y_{s1},y_{s2},...,y_{sns}\right]$, where $x_{si}$ is the DE feature of the *i*-th sample from the training set and $y_{si}$ is its corresponding class label. Similarly, feature vectors extracted from the test set are defined as the target domain and are denoted as $X_{T}=\left[x_{t1},x_{t2},...,x_{tnt}\right]$, where $x_{ti}$ is the DE feature of the *i*-th sample from the test set. Given a labeled source domain $X_{S}$ and unlabeled target domain $X_{T}$, the problem to be addressed is how to learn a new feature representation to reduce the marginal distribution discrepancy between $P_{S}\left(X_{S}\right)$ and $P_{T}\left(X_{T}\right)$, and the conditional distribution discrepancy between $P_{S}\left(Y_{S}|X_{S}\right)$ and $P_{T}\left(Y_{T}|X_{T}\right)$.

As shown in Figure 1, ASFM employs a two-step strategy. In the first step, the augmented DE feature $X_{S}$ in the source domain is aligned to the target domain along with its class label by using a fast marginal distribution adaptation strategy. In the second step, logistic regression is applied to the new source domain to train a classifier for use in the target domain. Then, the conditional distribution discrepancy is adapted by exploring the pseudo-labels of the target data. As a result, discrepancies in both marginal and conditional distribution between the source domain $X_{S}$ and unlabeled target domain $X_{T}$ can be reduced.

#### 3.2.1. Fast Marginal Distribution Adaptation

**Figure 3:**
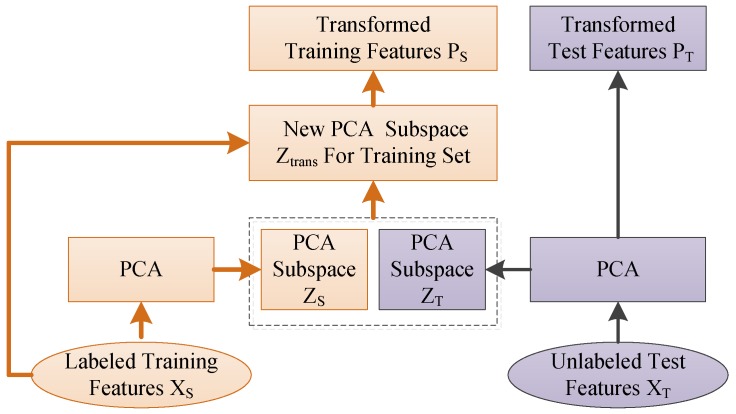
Architecture of the fast marginal distribution adaptation strategy. PCA: principal component analysis.

In order to match the marginal distribution of source and target domain, the subspace alignment (SA) algorithm proposed in literature [35] is used in this paper. For simplicity, as shown in Figure 3, principal component analysis (PCA) is chosen in this study for the selection of *d* eigenvectors corresponding to the *d* largest eigenvalues. These eigenvectors are used as the bases of the source and target subspaces, $Z_{S}$ and $Z_{T}$. The common domain adaptation strategies used to constrain the distribution discrepancy project both source and target data onto a common shared subspace by minimizing the maximum mean discrepancy (MMD). However, due to the very large amount of computation required, this strategy is unsuitable to be used for online classification. Thus, instead of learning the transformation from source and target data directly, the proposed method tries to find the linear transformation that best maps the source eigenvectors $Z_{S}$ onto the target eigenvectors $Z_{T}$.

Assuming that there exists a transformation matrix, *S*, which can align the coordinate systems of the transformed subspaces $Z_{S}$ and $Z_{T}$, the objective function can be expressed as:

(4) $$min({∥Z_{S}S-Z_{T}∥}_{F}^{2})$$

where ${∥\cdot ∥}_{F}^{2}$ is the Frobenius norm. Since the eigenvectors $Z_{S}$ and $Z_{T}$ extracted by PCA are orthonormal, $Z_{S}^{T}Z_{S}=I$ and $Z_{T}^{T}Z_{T}=I$, where *I* is the identity matrix. According to the invariance of orthonormal operations of the Frobenius norm, ${∥Z_{S}S-Z_{T}∥}_{F}^{2}$ can be re-written as:

(5) $$F\left(S\right)={∥Z_{S}^{T}Z_{S}S-Z_{S}^{T}Z_{T}∥}_{F}^{2}={∥S-Z_{S}^{T}Z_{T}∥}_{F}^{2}$$

Therefore, the best approximation is to assume that when $S^{*}=Z_{S}^{T}Z_{T}$, the objective function is a minimum. Given the source and target PCA subspaces $Z_{S}$ and $Z_{T}$, the transformed source subspace can be expressed as:

(6) $$Z_{Trans}=Z_{S}Z_{S}^{T}Z_{T}$$

By computing $X_{S}Z_{Trans}=X_{S}Z_{S}Z_{S}^{T}Z_{T}$ and $X_{T}Z_{T}$, the marginal distribution discrepancy between $P_{S}\left(X_{S}\right)$ and $P_{T}\left(X_{T}\right)$ can be reduced. Since only PCA and matrix multiplication are used in this transformation process, this method is very fast compared with other MMD-based domain adaptation methods [24,26].

#### 3.2.2. Conditional Distribution Adaptation

The above subspace feature-matching strategy is fast and effective; however, it still has one drawback in that although the marginal distribution discrepancy between source and target data has been reduced, the conditional distributions may also be mismatched due to changes in user attention level and user fatigue. The ASFM method, using a joint adaptation of conditional and marginal distributions, is therefore proposed to address this issue. Since there is a large difference between the marginal distributions of the training and test data, the problem of matching the source conditional distribution with the target is non-trivial. Thus, we adapt conditional distributions in the transformed subspace $X_{S}Z_{Trans}=X_{S}Z_{S}Z_{S}^{T}Z_{T}$ and $X_{T}Z_{T}$. Our goal is to match the conditional distributions $P_{S}\left(Y_{S}|X_{S}Z_{Trans};w\right)$ and $P_{T}\left(Y_{T}|X_{T}Z_{T}\right)$.

Denote $P=X_{S}Z_{Trans}$, and the transformed labeled source domain $D=\left\{\left(p_{1},y_{s1}\right),...,\left(p_{ns},y_{sns}\right)\right\}$. Logistic regression is used to estimate the conditional distributions of the source domain $P_{S}\left(Y_{S}|P;w\right)$.

The probability model of logistic regression is parameterized by a weight-vector *w* and defined as:

(7) $$P\left(y|p;w\right)=-\frac{1}{1+exp(-yw^{T}p)},where\;y=\pm 1$$

The gradient-descent algorithm is utilized to learn the parameter *w*. Then, the conditional distribution of the source domain can be defined according to Equation (7):

(8) $$P_{S}\left(Y_{S}=1|P;w\right)=P_{S}\left(Y_{S}=1|X_{S}Z_{Trans};w\right)=\frac{1}{1+exp(-w^{T}X_{S}Z_{Trans})}$$

However, $P_{T}\left(Y_{T}|X_{T}Z_{T}\right)$ cannot be exactly estimated without labeled data in the target domain. It is well known that labeling the sample is always time-consuming and requires specific conditions. To solve this problem, we assume that $P_{T}\left(Y_{T}|X_{T}Z_{T}\right)\approx P_{S}\left(Y_{S}|X_{S}Z_{Trans};w\right)$; thus, the pseudo-labels of the target data can be estimated. In order to achieve a more accurate approximation for $P_{T}\left(Y_{T}|X_{T}Z_{T}\right)$, we propose an iterative pseudo-label refinement strategy to refine $P_{T}\left(Y_{T}|X_{T}Z_{T}\right)$. In logistic regression, the probability $P_{S}\left(Y|X_{T}Z_{T};w\right)$ is a natural metric of the certainty that $X_{T}Z_{T}$ belongs to the predicted label. Based on this probability, the confidence level $q(X_{T}Z_{T})$, which is used to decide whether this target sample can be moved into the training set, can be defined as:

(9) $$q\left(Z_{T}\right)=\left\{\begin{array}{c} 1\;\\ 0\; \end{array}\right.\begin{array}{c} if\;P_{S}\left(Y|X_{T}Z_{T};w\right)>\tau \\ otherwise \end{array}$$

For EEG-based emotion recognition applications with a given training set *U* and a test set *V*, each new sample is assigned a confidence level *q*. If q=1, this sample will be moved into the training set *U*, and the conditional distribution $P_{S}\left(Y_{S}|X_{S}Z_{Trans};w\right)$ will be readjusted. As the number of iterations increases, $P_{S}\left(Y_{S}|X_{S}Z_{Trans};w\right)$ can be iteratively refined. Eventually, both the marginal distribution and the conditional distribution between domains will be reduced.

The pseudo code of our ASFM method is given in Algorithm 1.
**Algorithm 1** ASFM: adaptive subspace feature matching and classification.**Require:**  Source data $X_{S}$, Target data $X_{T}$, Source labels $Y_{S}$, Threshold τ, Subspace dimension *g*,  Maximum Iterations: maxepoch **Ensure:**   $Y_{T}$ are the predicted labels for Target data $X_{T}$.
 1:Calculate the source subspace: $Z_{S}\leftarrow PCA\left(X_{S},d\right)$. 2:Calculate the target subspace: $Z_{T}\leftarrow PCA\left(X_{T},d\right)$. 3:Align subspace from $Z_{S}$ to $Z_{T}$ to get new subspace: $Z_{Trans}=Z_{S}Z_{S}^{T}Z_{T}.$ 4:Project source data to its new subspace: $P_{S}=X_{S}Z_{Trans}$. 5:Project target data to its subspace: $P_{T}=X_{T}Z_{T}.$ 6:Initialize *w* randomly and learning rate α. 7:**for** epoch=1,2,...,maxepoch **do** 8: $w\leftarrow argmin\ell (w,\left(P_{S},Y_{S}\right))$. 9: Apply on all elements of $P_{T}$ by using Equation (8).10:Move *c* confident inputs from test set to the training set by using Equation (9).11:**end for**12:$Y_{T}\leftarrow classifier\left(P_{T}\right)$ by using Equation (8).


## 4. Experiment Results

In this section, an off-line experiment was first conducted on the SEED datasets to evaluate the efficacy of the proposed ASFM. Then, to show the computational efficiency of the proposed methods, a real-time experiment was also designed in this study. It should be noted that SEED consists of 15 participants and each one performed the experiments three times; the interval of two sessions is one week or longer. Therefore, we evaluate our ASFM model on two scenarios: (1) subject-to-subject evaluation, where the training and test domains are from different subjects; and (2) session-to-session evaluation, where the source and target domains are from the same subject but different experiment sessions.

### 4.1. Off-Line Evaluation

In some scenarios, such as psychological analysis and disease diagnosis, we need to record all of the samples in advance, then analyze the problem as a whole after data acquisition. Therefore, in order to evaluate the effectiveness of the proposed ASFM for reducing the marginal and conditional distribution discrepancy between training and test set, we first conduct an off-line experiment, in which all of the test samples were concentrated into one target domain, and classified simultaneously. We systematically compare the proposed ASFM model with several state of the art domain adaptation methods. Firstly, conventional support vector machine (SVM) and logistic regression (LR) without domain adaptation technology were trained as baseline by using Library for Support Vector Machines (LIBSVM) [36] and Library for Large Linear Classification (LIBLINEAR) [37], because we chose LR as the base classifier in our ASFM model. In addition, to testify the effectiveness of combining the marginal and conditional distribution adaptation strategies together, we design this comparison named “subspace feature matching (SFM)”. For SFM, we only match the marginal distribution by aligning the PCA subspace as shown in Section 3.2.1, without moving confident samples from test set to the training set. We systematically compared five state-of-the-art domain adaptation methods and subspace feature matching. It is worth mentioning that, for these domain adaptation methods, LR is chosen as the base classifier for fair comparison.

- Auto-encoder (AE) [28];
- Transfer sparse coding (TSC) [27];
- Transfer component analysis (TCA) [24].
- Transfer joint matching (TJM) [26];
- Subspace alignment auto-encoder (SAAE) [34];
- Subspace feature matching (SFM) without adaptive strategy;

In addition, the min-max strategy is used to normalize the EEG trials in this paper. EEG feature vectors extracted from the training and test set are denoted as $F_{S}$ and $F_{T}$. For the off-line evaluation, we concentrate $F_{S}$ and $F_{T}$ as $F=\left[F_{S}\;F_{T}\right]$, and our target is normalizing *F* to $\left[L,U\right]$. The formulation of the min-max normalization is:

(10) $$X=\frac{F-min(F)}{max(F)-min(F)}*\left(U-L\right)+L$$

where *X* is the normalized data, and *U* and *L* are the upper and lower normalization bounds. In this paper, *U* and *L* are as 1 and 0.

Table 1 shows the parameters of different methods. In this study, the SVM is employed by linear kernel, and the parameter C is set by searching {0.01, 0.05, 0.1, 0.5, 1, 5, 10, 50, 100} to find the optimal value. For LR, the parameter C was tuned in {0.01, 0.05, 0.1, 0.5, 1, 5, 10, 50, 100}. The structure of conventional auto-encoder was set with two hidden layers and the number of hidden neurons is set as 200. For TSC, the number of basis vectors in the dictionary was set to 150, sparsity regularization to 0.1, and the number of iterations per TSC to 15. Subspace learning method TCA and TJM were employed by radial basis function (RBF) kernel, and subspace bases were set as 80 and the adaptation regularization parameter λ is 1.0. For our adaptive subspace feature matching, the dimension of the PCA subspace is set to all, and threshold τ to 0.45, and the number of iterations is set to 1.

In this study, classification accuracy was used as the evaluation metric [26]:

(11) $$Accuracy=\frac{\left|\begin{array}{c} X:X\in D_{T}\wedge \hat{Y}\left(X\right)=Y\left(X\right) \end{array}\right|}{\left|\begin{array}{c} X:X\in D_{T} \end{array}\right|}$$

where $D_{T}$ is the set of test data, Y(X) is the truth label of *X*, and $\hat{Y}\left(X\right)$ is the label predicted by the classification algorithm.

#### 4.1.1. Subject-To-Subject Experimental Results

To confirm the effectiveness and stability of our method, the leave-one-subject-out cross validation method was adopted for the evaluation. There are in total 15 subjects in the SEED database. It is worth mentioning that it is infeasible to include all the data (about 47,516 samples) from 14 subjects as the training data for TSC, TCA, and TJM because of the limits of the memory for singular value decomposition [22]. There are in total 15 trials for each subject in one experiment. Therefore, to avoid bias, we randomly selected a 20 samples from each trial and got 300 samples in total. That means the original structure of the SEED dataset is retained. Ultimately, from each subject, we obtained 4200 samples in total as training data. In addition, we repeated our sample selection five times to get a fair result. For a fair comparison, all of the methods were evaluated by using the same 4200 samples as the training set in this paper.

Table 2 shows the average classification accuracy and standard deviations of the 15 subjects for each experimental session. It is worth noting that the average standard deviation is the standard deviation for the 15 participants after calculating the average accuracy of three sessions. As seen from these comparisons, the two standard classifiers SVM and LR only achieve an average classification accuracy and standard deviation of 57.29%/7.42% and 57.43%/7.95%, respectively, as no domain adaptation strategy is employed. With the benefits of learning common representations from both source and target domains by utilizing the deep structure, the auto-encoder method can achieve the mean and standard deviation of accuracy of 61.71%/8.09%, respectively, which is slightly better than SVM, and LR. TSC, which attempts to modify the representation of both source and target data, outperformed the auto-encoder method, benefiting from sparse coding constraint. As expected, TCA and TJM, which use single-kernel MMD to match different distributions, achieved much better performance at 75.32%/11.07% and 76.24% and 10.38%, respectively, as both of them try to minimize the distribution divergence with a certain constraint. The SAAE method, which utilizes the nonlinearity of the auto-encoder coupled with MMD constraints to ensure that the shifted source domain, follows a distribution similar to that of the target domain, and achieved a better accuracy performance at 77.88%/7.33% for mean and standard deviation, respectively. Compared with these method, the average classification accuracy and standard deviation of our ASFM is 80.46%/6.84%, respectively, gaining a 2.58% higher accuracy and 0.49% lower standard deviation compared to the best baseline SAAE, and 2.83%/−1.00% compared to SFM. In addition, compared with the subject-to-subject transfer approach proposed by Zheng et al. [22], also evaluated on the SEED dataset, our ASFM method achieves 80.46%/6.84% for mean and standard deviation, better than the 76.31%/15.89% for mean and standard deviation found in the literature [22]. The statistical significance of the performance between the proposed ASFM approach and other algorithms is evaluated using Student’s *t*-test, and the *p*-values is lower than 0.05 for all tests.

#### 4.1.2. Session-to-Session Experimental Results

One of the great advantages of SEED dataset is that each participant was required to perform the experiments for three sessions, and the interval lasts at least one week. This can help us investigate whether the performance of our domain adaptation method can overcome the non-stationarity of EEG signals with the passage of time. In this study, six task groups were constructed by splitting the data from different sessions of one subject to training data and test data: session 1 → session 2, session 1 → session 3, session 2 → session 1, session 2 → session 3, session 3 → session 1, and session 3 → session 2. For each task group, all the data from one session (about 3394 samples) were included as the training data, and the data from another session (about 3394 samples) were included as the test data.

Table 3 shows the mean accuracy and standard deviations of our ASFM method and the other nine baseline methods on six task groups. It is worth mentioning that Graph regularized Extreme Learning Machine (GELM) [38], which has been validated as the most effective method for the session-to-session task on SEED dataset, is added for comparison. The reported results in the original paper were used for a fair comparison. We observed that GELM achieved a much better performance compared to the best baseline SVM and LR, as they forced the output of samples from the same class to be similar. As expected, TCA and TJM achieved much better performance at 80.68%/7.70% and 81.56%/7.47% for mean accuracy and standard deviation. The SAAE method achieved a better accuracy and standard deviation performance, at 81.81%/7.56%. The average classification accuracy and standard deviation of ASFM on the session-to-session task is 84.61%/6.92%, gaining a significant performance improvement of 2.80%/0.64% compared to the best baseline SAAE. The statistical significance of the performance between the proposed ASFM approach and other algorithms is evaluated using Student’s *t*-test, and the *p*-values is lower than 0.05 for all tests.

### 4.2. On-Line Evaluation

**Figure 4:**
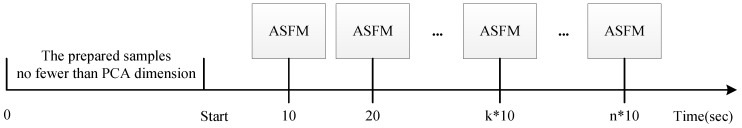
The flow of online experiment.

To verify the effectiveness of our ASFM method for real-time classification, a simulated on-line experiment was designed in this section. In the on-line testing evaluation, a conventional LR classifier was trained by all training data, then about 3394 epochs (each epochs lasts for 1 s), were tested one by one as time progressed. Compared to the fixed decision rule based classification method, our ASFM method is adaptively performed for each test data. It is worth mentioning that the amount of samples in target domain must be greater than the subspace dimension *d* for PCA processing. Therefore, as shown in Figure 4, when the amount of samples in target domain is greater than the subspace dimension *d*, we can use ASFM for online applications. In addition, since the distribution of the test domain may always fluctuate, it is inappropriate to train a fixed classifier to classify the rest of the following samples. Therefore, in this paper, the classifier was adjusted every 10 s by our ASFM method as shown in Figure 4. The training set is denoted as $D_{S}$ and the test set is denoted as $D_{T}$. Our strategy is that we add 10 new samples into $D_{T}$, and use $D_{S}$ and $D_{T}$ as the input of our ASFM model to learn a new classifier every 10 s. As time goes on, both marginal and conditional distribution discrepancy between training and test sets can be reduced along with this inherent adaptive characteristic of the ASFM.

The min-max strategy is used to normalize the EEG trials in this paper. EEG feature vectors extracted from the training and test set are denoted as $F_{S}$ and $F_{T}$. For the on-line evaluation, test samples were normalized according to the minimal and maximal values defined by the training samples only. The formulation of the min-max normalization is:

(12) $$X=\frac{F_{T}-min\left(F_{S}\right)}{max\left(F_{S}\right)-min\left(F_{S}\right)}*\left(U-L\right)+L$$

where *X* is the normalized data, and *U* and *L* are the upper and lower normalization bound. In this paper, *U* and *L* are as 1 and 0.

Because of the high time complexity, the previously mentioned domain adaptation methods, such as TJM, TCA, AE, and TSC, are not suitable for online use. Therefore, in this study, we only compare the classification performance of the proposed ASFM method with the conventional SVM and LR. Table 4 shows the parameters of different methods. Conventional support vector machine (SVM) and logistic regression (LR) without domain adaptation technology were trained as baseline by using LIBSVM software [36] and LIBLINEAR software [37], because we chose LR as the base classifier in our ASFM model. In this study, the SVM is employed by linear kernel, and the parameter C is set by searching {0.01, 0.05, 0.1, 0.5, 1, 5, 10, 50, 100} to find the optimal value. For LR, the parameter C was tuned in {0.01, 0.05, 0.1, 0.5, 1, 5, 10, 50, 100}. For our adaptive subspace feature matching, the dimension of the PCA subspace was set to all, and threshold τ to 0.41, and the number of iterations was set to 1.

**Figure 5:**
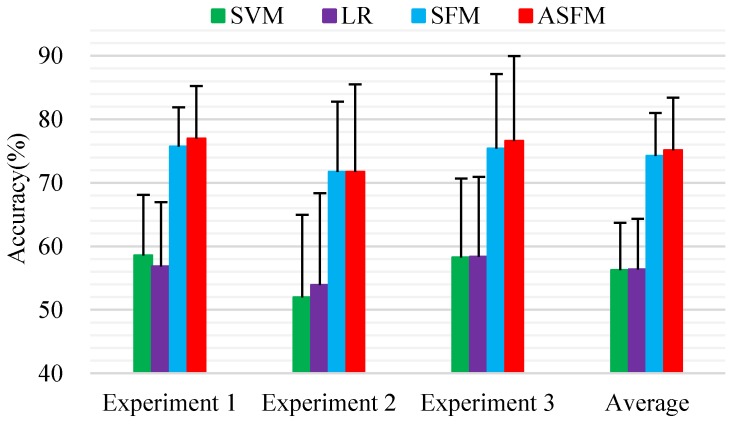
Average classification accuracy and standard deviations (%) of each subject using leave-one-subject-out cross validation method for on-line evaluation.

As mentioned before, the leave-one-subject-out cross validation method was adopted for the evaluation. To reduce the computational complexity, a subset of samples (300 samples) was randomly selected from each subject, and 4200 samples were obtained in total as training data. All the data from each subject (about 3394 samples) were included as the test data. To get a fair result, we repeated our sample selection five times, then average classification accuracy was used as the final classification outcome. Figure 5 demonstrates the detailed classification accuracy and standard deviations of all the applicable methods. We note that SVM and LR achieved comparable performances of 56.26%/6.84% and 56.38%/7.48% for mean and standard deviation. The average classification accuracy and standard deviations of ASFM on the session-to-session task is 75.11%/7.65%, gaining a significant performance improvement compared to the best baseline LR. It is very important to observe that ASFM performs better than SFM (74.26%/6.19% for mean accuracy and standard deviation), which is our subspace feature matching model without adaptive strategy on this dataset. This reveals that only matching the marginal distribution may not be good enough for robust domain adaptation.

**Figure 6:**
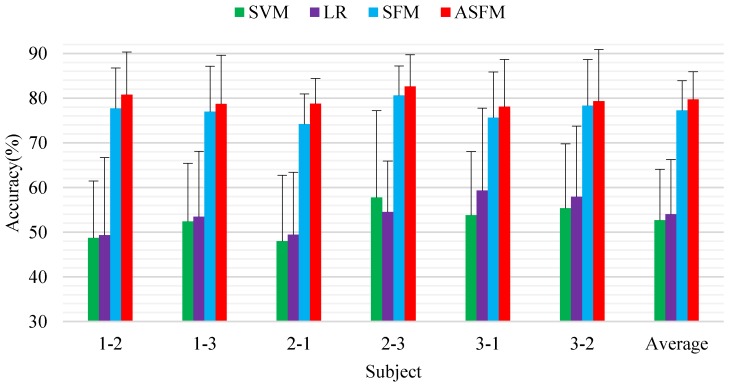
Average classification accuracy and standard deviations (%) of training and test data from different sessions for on-line evaluation.

Session-to-session experiment was also performed for online test. Six task groups were constructed by splitting the data from different sessions of one subject to training data and test data: session 1 → session 2, session 1 → session 3, session 2 → session 1, session 2 → session 3, session 3 → session 1, and session 3 → session 2. For each task group, all the data from one session (about 3394 samples) were included as the test data, and the data from another session (about 3394 samples) were included as the test data. Figure 6 demonstrates the detailed experimental results.

As seen from these comparisons, the two standard classifiers SVM and LR only achieve an average classification accuracy and standard deviation of 52.68%/5.63% and 54.01%/6.29% as no domain adaptation strategy is employed. The average classification accuracy and standard deviation achieved by ASFM on the six tasks were 79.71%/4.97% and performed better than SFM (77.24%/5.32%). This verifies that the proposed models can overcome the non-stationarity of EEG signals over time. The statistical significance of the performance between the proposed ASFM approach and other algorithms is evaluated using Student’s *t*-test, and the *p*-values is lower than 0.05 for all tests.

## 5. Discussion

### 5.1. Comparison with State-of-the-Art Methods

To give a better understanding of the comparison results, in this section, we discuss the algorithmic differences of these state-of-the-art methods. SVM and LR are known to perform fairly well on standard emotion recognition classification problems, but it may fail when the training data and testing data are sampled from different domains.

For the stacked auto-encoder, the nonlinearity of the deep structure made it feasible to extract common representations from both source and target domains. However, without consistency constraints, it is not always optimal because the new deep features may unexpectedly increase the distribution discrepancy at times. Domain adaption methods, such as TSC, TCA and TJM, have been successfully applied for reducing the cross-domain discrepancy in the field of computer vision. However, for EEG sensor data, they did not achieve the best performance. The sparse coding based algorithm TSC creates new representations by incorporating MMD into the objective function to constrain the cross-domain discrepancy. TCA transforms features into a subspace with MMD constraints. As an improved method of TCA, TJM aims to reduce the domain difference by jointly matching the features and reweighting the instances across domains by constructing invariant feature representation. For a computer vision task, there are a lot of irrelevant features among different images. Therefore, transforming features into a common shared subspace is an effective way to reduce distribution discrepancy and the irrelevant features. However, for EEG sensor data, projecting the data to an intermediate common shared subspace may lead to information loss in both source and target domains, because most features extracted from all the 62 channels are significant. Moreover, such a strategy is costly, and renders them unsuitable for real-time classification. The experimental results shown in Section 4 demonstrate this drawback.

As compared with the other algorithms, our ASFM method projects each source and target data into its respective subspace, then learns a transformation matrix to make the coordinate system of the transformed subspaces align. As a linear transformation, ASFM still cannot fully remove the distribution discrepancy completely, but most information of each domain can be retained. Furthermore, for our ASFM, both marginal and conditional distribution discrepancy are taken into account. The experimental results in Section 4 have shown that ASFM performed better than SFM, which is our subspace feature matching model without matching the conditional distribution. This significantly demonstrates the effectiveness of our ASFM method.

### 5.2. Computation Time Analysis

As already mentioned, a simulated on-line experiment was designed in Section 4.2. In fact, as the test samples increase, the computation time for adjusting the classifier also increased. In order to avoid the bias, we only evaluated time complexity when we get the maximum quantity of the test samples (4200 training samples and 3394 test samples.) following the on-line experiment designed in Section 4.2. It is worth mentioning that the time taken for data preprocessing and feature extraction was not considered. All the algorithms were tested in the same environment using MATLAB R2013b with an Intel core i7-4770 3.40 GHz processor and 16 GB of RAM. The experimental results are shown in Table 5.

**Figure 7:**
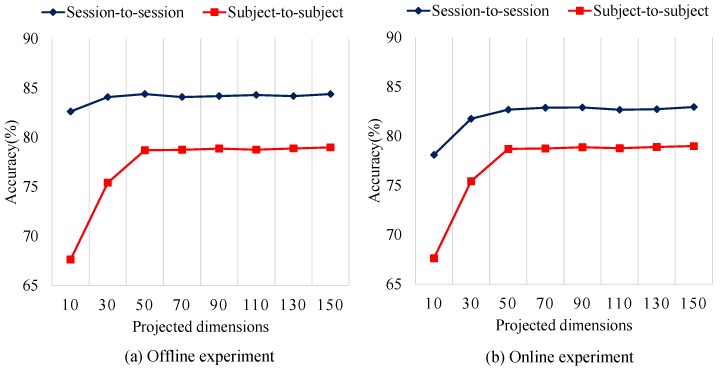
Average classification accuracy of the ASFM method with varying subspace dimension.

With no surprise, the computation time of the SVM and LR is much shorter than the other algorithms. All of the domain adaptation methods (AE, TSC, TCA, TJM) need a lot of training time, and this renders them un-suitable for real-time classification. Our ASFM method achieves a acceptable performance in this computation time experiment. Compared to SFM, the adaptive strategy really needs extra time. It is worth mentioning that the time complexity of our ASFM is closely related to the subspace dimension. In Figure 7 and Table 6, the average classification accuracies and time complexity of our ASFM method with varying subspace dimension are shown.

We also analyze the computational complexity of Algorithm 1 using the big *O* notation. The computational cost focuses primarily on PCA (Line 3 and Line 4) and parameter estimation for (Line 8). In this paper, if there are $n_{1}$ training samples and $n_{2}$ test samples, each represented with *m* features, then, for PCA: covariance matrix computation is $O(m^{2}\left(n_{1}+n_{2}\right))$ and its eigen-value decomposition is $O(m^{3})$. Therefore, the complexity of PCA is $O(m\left(m\left(n_{1}+n_{2}+m\right)\right))$. The complexity of parameter estimation for *w* is determined by parameter estimation method. In this paper, the random gradient-descent algorithm is utilized in this learning work, and the complexity is $O(m\left(n_{1}+n_{2}\right))$. Authors of [24] showed that the complexity of comparison method TCA is $O(m{\left(n_{1}+n_{2}\right)}^{2})$. In this paper, $n_{1}+n_{2}=7594$ and m=325. Therefore, complexity of TCA is about 20 times more than our method. This is similar with the results shown in Table 5.

It is worth noticing that the proposed ASFM method has achieved balanced results when 70 features are extracted. In spite of this, complexity of our method is still limited by the number of training samples and test samples. In order to use our method for online implementation, the number of training samples and test samples should be paid attention, for example, by enlarging the sampling interval of the EEG features when necessary.

### 5.3. Effectiveness Analysis

**Figure 8:**
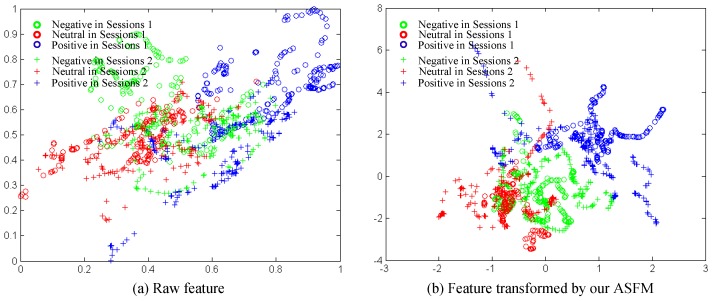
Feature distributions of the first two dimensions between training and testing sessions for Subject 1.

To prove the effectiveness of the proposed methods, we show scatter plots of features between the training dataset (session 1) and the testing dataset (session 2) for Subject 1. To facilitate visualization, we utilize the first two dimensions to plot the feature distribution. In Figure 8, the red, green and blue plus sign marks indicate the training features from Session 1. On the other hand, the red, green and blue circles indicate the test features from Session 2. Therefore, it is easy for us to check the distribution change from the training to the test data during the experimental sessions. Figure 8a shows the distribution of raw EEG features. Obviously, a different feature distribution is found to occur. Figure 8b shows that the features from different sessions have been transformed into a subspace by our ASFM method. We can observe that the target points in Figure 8b are discriminated much better, and the categories between the source and target are aligned much better. Clearly, the domain discrepancy of the two sessions has been significantly reduced, and it can explain the effectiveness of our ASFM.

### 5.4. Online Trajectory Analysis

**Figure 9:**
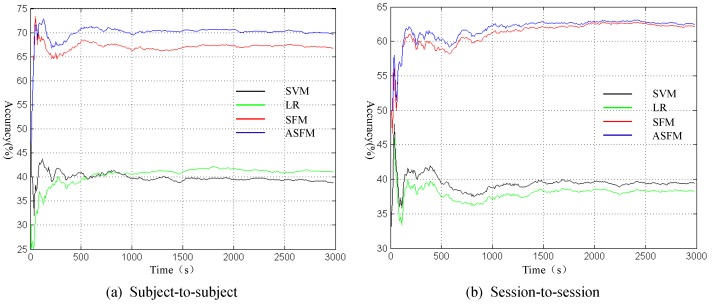
The profile of accuracy varying with time in session-to-session and subject-to-subject experiment for Subject 1.

To analyze the online trajectory, we draw the profile of accuracy varying with time along the currently adopted temporal resolution of 10 s. Due to space limitations, the session-to-session and subject-to-subject experiment for Subject 1 are shown in Figure 9 as an example. In Figure 9, the black, green, red and blue curves indicate SVM, LR, SFM, and ASFM. As can be observed from Figure 9, all of the methods show an unstable results in the early stages, because the quantity of test sample is too small. As time progresses, the results reach stability gradually. It is worth noting that our ASFM strategy almost gains the best performance at all time-points.

### 5.5. Parameter Sensitivity

In this study, the proposed ASFM method involves two important parameters: the dimensionality of the subspace *k*, and the threshold τ, which determines the confidence level $q(X_{T}Z_{T})$. In the previous section, we have shown the average classification accuracies and time complexity of our ASFM method with varying subspace dimension in Figure 7 and Table 6. Taking account of time complexity, in this study, the dimensionality of the subspace *k* was set as 70.

The threshold τ is another important parameter for ASFM. We conducted sensitivity analysis on the leave-one-subject-out cross validation experiment with 325 features, 4200 training samples and 3394 test samples. For comparison, the average classification accuracy of all 15 task groups on the subject-to-subject datasets was computed. When testing this specific parameter, the dimensionality of the second subspace was fixed at 70. The influence of the parameter τ in our ASFM approach is shown in Table 7. The threshold τ should be neither too small nor too large. In this study, for all experiments, the threshold τ of ASFM was set at 0.45.

In addition, we added analysis about the influence of the number of iterations in our ASFM approach. Table 8 shows classification accuracy of ASFM using different number of iterations. It can be observed that as the number of iterations increases, classification accuracy stabilizes gradually. However, there is only a small increase when the number of iterations is more than one. Therefore, taking account of time complexity, we only use one time iteration.

### 5.6. The limitations of ASFM

The limitations of ASFM are mainly reflected in the following two aspects:

(1) As some works [39,40,41] have mentioned, although there are many successful applications, the power of transfer learning still has its limit, which is named “negative transfer”. Negative transfer refers to the phenomenon that, instead of improving performance, transfer learning from source domain degrades the performance on the target domain. In general, transfer learning methods treat knowledge from every source domain as a valuable contribution to the task on the target domain. However, when knowledge is transferred from highly irrelevant sources, “negative transfer” will happen. In fact, given multiple source domains, in the worst case, the majority of the source domains could be irrelevant to the target domain. In reality, ASFM is not effective on DEAP [32] which is a frequently-used database for EEG-based emotion estimation. This is probably because there are too many irrelevant subsets between each other in this dataset. So far, it is still difficult to estimate the relevance between two subjects without labeled data in the target subject. Therefore, unsupervised transfer learning (or domain adaptation) research for EEG-based emotion estimation on DEAP database is a greater challenge, and an important research aspect in our future work.
(2) The complexity of our method is still limited by the number of training samples and test samples. In order to use our method for online implementation, the number of training samples and test samples should be paid attention, for example by enlarging the sampling interval of the EEG features when necessary.

## 6. Conclusions

In this paper, the ASFM scheme is proposed, which attempts to jointly adapt both the marginal and conditional distributions from non-stationary EEG data across different subjects and sessions. In ASFM, linear mapping is learned in order to align the source PCA subspace with the target PCA subspace. Following this, conditional distributions can be adapted in the transformed subspace by iterative exploration of the pseudo-labels of the target data. As a result, discrepancies in both marginal and conditional distributions between the source domain and the unlabeled target domain can be reduced. Experimental testing using the SEED dataset for EEG emotion recognition shows that the proposed method demonstrates improved performance compared with the state-of-the-art methods, and verifies the effectiveness of the proposed method for dealing with the domain discrepancy and reducing performance degradation across subjects and sessions for offline and real-time applications.

## Figures and Tables

**Table 1:** Details of parameters used in different methods. SVM: support vector machine; LR: logistic regression; AE: auto-encode; TSC: transfer sparse coding; TCA: transfer component analysis; TJM: transfer joint matching.

Method	Parameter Details
SVM	Linear kernel; the parameter C was set by searching $\left\{0.01,0.05,0.1,0.5,1,5,10,50,100\right\}$;
LR	L2-regularized logistic regression; We set the parameter C by searching $\left\{0.01,0.05,0.1,0.5,1,5,10,50,100\right\}$;
AE	Structure with 2 hidden layers; The number of hidden neurons was set by searching $\left\{50,100,200,400,600,1000\right\}$;
TSC	Number of basis vectors was 150; Sparsity regularization was 0.1; Number of iterations per TSC was set as 15;
TCA	Subspace bases was set as 80; Regularizer was set by searching $\lambda \in \left\{0.01,0.1,1,10,100\right\}$;
TJM	Subspace bases was set as 80; The number of iterations for TJM to converge is T = 3. Regularizer was set by searching $\lambda \in \left\{0.01,0.1,1,10,100\right\}$;
ASFM	The dimension of the PCA subspace is set to all; Threshold τ is set to 0.45; the number of iterations is set to 1.

**Table 2:** Average classification accuracy and standard deviations (%) of the 15 subjects for each experimental session using leave-one-subject-out cross validation method. The reported results from the original papers are marked with *. SAAE: subspace alignment auto-encoder; SFM: subspace feature matching. (bold numbers indicate the best results).

Task Group No.	Experimental Session 1	Experimental Session 2	Experimental Session 3	Average
SVM	60.31/9.53	53.37/12.97	58.20/12.40	57.29/7.42
LR	59.84/10.12	54.32/14.42	58.12/12.58	57.43/7.95
AE [28]	65.64/10.44	55.82/11.52	63.67/12.31	61.71/8.09
TSC [27]	73.33/7.61	72.16/9.08	67.03/9.79	70.84/6.44
TCA [24]	75.91/11.52	74.19/12.26	75.87/6.87	75.32/11.07
TJM [26]	77.62/10.90	74.30/12.06	76.79/5.69	76.24/10.38
SAAE * [34]	80.22/8.00	74.68/12.76	78.73/12.96	77.88/7.33
SFM	80.34/6.13	74.81/10.51	77.74/10.15	77.63/5.84
ASFM	83.51/7.40	76.68/12.16	81.20/10.68	80.46/6.84

**Table 3:** Average classification accuracy and standard deviations(%) of training and test data from different sessions. The reported results from the original papers are marked with *.(bold numbers indicate the best results).

Task Group No.	1→2	1→3	2→1	2→3	3→1	3→2	Average
SVM	67.63/12.73	64.21/13.00	67.62/14.73	70.37/19.48	72.69/14.21	66.64/14.38	68.19/11.41
LR	60.64/17.38	60.42/14.61	58.82/13.97	65.30/11.37	63.89/18.44	63.85/15.79	62.15/9.51
GELM * [38]	72.55/10.29	67.22/10.42	75.86/7.71	76.62/15.34	76.28/11.47	78.17/13.41	74.45/8.20
AE [28]	76.66/8.92	75.30/10.83	77.47/11.54	77.20/15.35	77.02/12.81	78.21/13.15	76.98/9.52
TSC [27]	79.85/12.12	80.71/10.70	82.07/8.08	80.24/10.32	79.92/7.71	77.99/11.36	80.13/8.52
TCA [24]	81.56/11.52	79.35/12.26	81.56/6.87	82.83/11.07	80.84/8.00	77.97/13.90	80.68/7.70
TJM [26]	82.43/10.90	80.89/12.06	82.36/5.69	84.04/10.38	80.57/9.97	79.09/11.69	81.56/7.47
SAAE * [34]	84.47/10.06	80.04/13.03	84.31/7.21	83.09/9.98	80.20/9.99	78.77/10.49	81.81/7.56
SFM	83.09/10.12	81.61/10.42	84.11/6.23	84.21/8.19	84.62/9.59	82.16/11.06	83.30/7.12
ASFM	84.34/10.18	82.96/10.49	85.46/6.07	85.11/8.83	86.05/9.66	83.75/11.39	84.61/6.92

**Table 4:** Details of parameters used in different methods.

Method	Parameter Details
SVM	Linear kernel; the parameter C was set by searching $\left\{0.01,0.05,0.1,0.5,1,5,10,50,100\right\}$;
LR	L2-regularized logistic regression; we set the parameter C by searching $\left\{0.01,0.05,0.1,0.5,1,5,10,50,100\right\}$;
SFM	The dimension of the PCA subspace is set to all.
ASFM	The dimension of the PCA subspace is set to all; Threshold τ is set to 0.41; The number of iterations is set to 1.

**Table 5:** Time complexity of all baseline methods. The reported results from the original papers are marked with *.

	SVM	LR	AE	TSC	TCA	TJM	SAAE *	SFM	ASFM
Training time (*s*)	0.20	0.29	90.14	246.39	58.08	164.92	121.81	0.39	1.39

**Table 6:** Time complexity of ASFM with varying subspace dimension.

Subspace Dimension	10	30	50	70	90	110	130	150	250	325
Training time (*s*)	0.19	0.26	0.30	0.37	0.45	0.50	0.58	0.63	1.01	1.39

**Table 7:** Classification accuracy (%) of ASFM using different values of the threshold τ.

τ	0.1	0.2	0.3	0.4	0.45	0.5	0.6	0.7	0.8	0.9
Classification accuracy (%)	77.45	77.48	78.23	79.49	80.46	79.73	78.31	77.95	77.82	77.71

**Table 8:** Classification accuracy (%) of ASFM using different number of iterations.

Number of Iterations	0	1	2	3	4	5	6	7
Classification accuracy (%)	77.63	80.46	80.69	80.90	81.05	81.08	81.09	81.09
